# The targeting of tumor-associated macrophages by vaccination

**DOI:** 10.15698/cst2019.05.185

**Published:** 2019-04-24

**Authors:** Mads Hald Andersen

**Affiliations:** 1 National Center for Cancer Immune Therapy (CCIT-dk), Copenhagen University Hospital, Herlev, DK-2730 Herlev, Denmark.; 2 Department of Immunology and Microbiology, University of Copenhagen, DK-2200 Copenhagen, Denmark.; 3 IO Biotech ApS, DK-2200 Copenhagen, Denmark.

**Keywords:** anti-Tregs, immune modulatory vaccines, IDO, arginase, PD-L1, CCL22, T-win

## Abstract

Many different therapeutic strategies focus on targeting tumor-associated macrophages (TAMs), due to their vital role in creating an immune suppressive tumor microenvironment (TME) with the aim to deplete, repro-gram or target the functional mediators secreted by these cells. Immune modulatory vaccination is an emerging strategy to target immune suppressive myeloid populations in the TME. In contrast to the other clinical strategies that target TAMs, this combines both TAM depletion (through direct killing by cytotoxic T cells) and TAM reprogramming (by introducing pro-inflammatory cytokines into the immune suppressive microenvironment).

Immune suppressive myeloid cells include a heterogenous population of cells that include tumor-associated macrophages (TAMs). TAMs are major players in inducing the immunosuppressive microenvironment associated with many tumors. These lesions were recently defined as tumors with an “excluded” phenotype, because they excluded CD8(+) T cells from the tumor parenchyma [1]. The infiltration of TAMs in tumors was correlated with a poor prognosis and a poor response to therapies, including checkpoint inhibitor therapies [2]. Consequently, TAMs are important therapeutic targets. Many studies have focused on anti-TAM strategies that aimed to deplete or reprogram these cells or target the functional mediators secreted by these cells [3]. These strategies should reverse tumor resistance to conventional therapies and promote response to T-cell-based therapies.

Immune modulatory vaccination is a novel unorthodox way of targeting the cancer-associated myeloid cell populations in the tumor microenvironment (TME). Anti-regulatory T cells (anti-Tregs) are defined as cells that can specifically react to regulatory immune cells, including TAMs, and restrict the range of immunosuppressive signals mediated by such cells [4, 5]. Anti-Tregs recognize HLA-restricted epitopes of proteins, including indoleamine 2,3-dioxygenase (IDO), arginase, and PD-L1. Activated anti-Tregs can revert the TME into an immune permissive site. The first clinical testing of IDO vaccinations was performed in patients with non-small-cell lung carcinoma (NSCLC; NCT01543464) [6]. Interestingly, although it was a small, phase I trial, vaccinated patients had a median overall survival of 26 months, which was significantly longer (P=0.03) than untreated control patients (median overall survival, eight months). Furthermore, two of 15 patients were long-term responders, with a clinical response that has continued for six years after the first vaccination, without any other treatment [7]. An ongoing industry-sponsored multi-centered phase II clinical trial was recently initiated to test IDO vaccinations in combination with pembrolizumab as a first-line treatment for patients with NSCLC (NCT03562871). Additionally, a phase I PD-L1 based vaccination in multiple myeloma (NCT03042793) is ongoing, and a phase I/II trial that targeted both IDO- and PD-L1-specific T cells in combination with Nivolumab is running in metastatic melanoma (NCT03047928). To date, these vaccinations have been well tolerated in all patients, with no grade III or IV toxicity. Finally, a phase I vaccination trial with arginase peptides was recently initiated at our institution (NCT03689192).

An important consideration related to therapeutic immune modulatory vaccines are the activation of both CD8 and CD4 anti-Tregs. Current cancer vaccines strategies targeting neo-antigens aim to induce cancer-specific CD8 cytotoxic T cells. However, in contrast to cancer vaccines the activation of anti-Tregs has the purpose to convert an immunosuppressive environment into a pro-inflammatory environment. TAMs are not terminally differentiated cells and they may be reverted into M1 macrophages given a pro-inflammatory stimulus. CD4 cells are the most compelling cytokine-producing cells. Consequently, CD4 anti-Tregs may be at least as important to activate as CD8 anti-Tregs in a therapeutic setting. Thus, in contrast to the other clinical strategies targeting TAMs, utilizing anti-Tregs combine both TAM depletion (by direct killing by cytotoxic T cells) as well as TAM reprogramming (by providing pro-inflammatory cytokines into the immune suppressive microenvironment). Both may be vital in the rebalance of the microenvironment, which should increase the effect of T-cell enhancing drugs, e.g. checkpoint blockers, as infiltration of TAMs in the TME is a major reason for the limited effect of checkpoint blockers in most patients with cancer. Therapeutic vaccines that activate anti-Tregs attract T cells into the tumor, inducing Th1 inflammation, and in turn further inducing proteins like IDO and PD-L1 in cancer, immune, and stromal cells. This activity would generate targets more disposed to anti-PD1/PDL1 immunotherapy. Hence, immune modulatory vaccines that the rebalance of the microenvironment should increase the effect of T cell–enhancing drugs such as checkpoint blockers. Combination therapy with immune modulatory vaccines and checkpoint blocking antibodies should therefore increase the number of patients who could respond to therapy.

In conclusion, immune modulatory vaccines have emerged as an alternative approach for directly targeting TAMs in the TME, compared to traditional antibodies or small molecule inhibitors.
